# Relationship between clinical findings and genetic mutations in patients with familial Mediterranean fever

**DOI:** 10.1186/s12969-015-0057-1

**Published:** 2015-12-12

**Authors:** Ayse Kilic, Muhammet Ali Varkal, Mehmet Sait Durmus, Ismail Yildiz, Zeynep Nagihan Yürük Yıldırım, Gorkem Turunc, Fatma Oguz, Mujgan Sidal, Rukiye Eker Omeroglu, Sevinc Emre, Yasin Yilmaz, Fatih Mehmet Kelesoglu, Genco Ali Gencay, Sonay Temurhan, Filiz Aydin, Emin Unuvar

**Affiliations:** Department of General Pediatrics, Istanbul University, Istanbul Medical Faculty, 34090 Istanbul, Turkey; Department of Pediatric Nephrology, Istanbul University, Istanbul Medical Faculty, Istanbul, Turkey; Department of Pediatrics, Istanbul University, Istanbul Medical Faculty, Istanbul, Turkey; Department of Pediatrics, Istanbul University, Institute of Child Health, Istanbul, Turkey; Department of Pediatric Rheumatology, Istanbul University, Istanbul Medical Faculty, Istanbul, Turkey; Department of Medical Biology, Istanbul University, Istanbul Medical Faculty, Istanbul, Turkey

## Abstract

**Background:**

Familial Mediterranean fever (FMF) is one of the most frequent genetic diseases encountered in the Mediterranean region. We aimed to investigate the correlation between genetic mutations and the clinical findings in 562 patients with FMF.

**Methods:**

In this retrospective cross-sectional study conducted with patients’ files between 2006, and 2013, reverse hybridization assay for MEFV gene mutations was used and the 12 most frequent mutations were screened. Mutation types and clinical findings were compared with variance analysis.

**Results:**

The mean age was 6.9 ± 3.4 years (range, 1.8-11.6 years). The most common symptom was fever (97.3 %). Thirty-four of the patients (6.04 %) were admitted with periodic fever only. Of these patients, M694V was the most common mutation type (73.5 %). The percentage of the patients predominantly presenting with recurrent abdominal pain was 77.78 % and the most frequent mutations were M694V and E148Q. The rate of arthritis and arthralgia was significantly higher in patients with M694V and E148Q mutations. Chest pain was reported more often in patients homozygous for M694V (61.4 %). Pericardial effusion was documented in the echocardiography of 10.9 % of the 229 children with chest pain. Some patients had both FMF and Henoch Schönlein purpura (HSP), and were more likely to harbor either homozygote M694V or E148Q mutations. The frequency of episodes was higher in patients with homozygous M694V mutations (number of attacks = 4.4 ± 1.6/month). Proteinuria was detected in 106 patients of cases (29.2 %), at an average of 854 ± 145 mg/L. Most of the patients with proteinuria and elevated serum amyloid-A had homozygous M694V mutation.

**Conclusion:**

The most common mutation in children in Turkey with FMF is the M694V mutation. Recurrent abdominal pain, arthritis or arthralgia, chest pain, and pericarditis were commonly seen in patients with M694V and E148Q mutations.

## Background

Familial Mediterranean Fever (FMF) is the most frequent autoinflammatory disease in Turkey [1, 2]. It is commonly seen among Turkish, Armenian, Arabic and Sephardic Jewish populations [3].

The most noticable finding of the disease is painful febrile episodes accompanied by peritonitis, pleuritic pain or acute synovitis in many patients. Headache or diffuse myalgia and malaise can also occur. The acute phase reactants like serum amyloid A (SAA) are significantly elevated [4, 5]. Recurrent orchitis and meningitis are uncommon presentations.

To date, many mutations on the responsible pyrin gene have been reported [6]. The pyrin gene was mapped on the short arm of chromosome 16 and contains approximately 60 kb sized genomic DNA [7].

In trials conducted in Turkish population, the most common mutations were reported as M694V, E148Q, M680I and V726A [8]. The last ten years have seen much research into the effect of particular genetic mutations on clinical findings [9].

In this study, we aimed to investigate the correlation between genetic mutations and clinical findings on admission of 562 patients with recurrent fever, abdominal pain and/or arthritis/arthralgia and subsequently diagnosed as having FMF. The relationship between genetic mutations, types and the severity of clinical findings in patients who were diagnosed as having FMF were analyzed. Furthermore, the relationship between frequency of proteinuria and genetic mutations were studied and the effect of colchicine on SAA was demonstrated.

## Methods

### Study group

We conducted a retrospective cross-sectional study. Data were transferred from the hospital software and patients’ files of patients who were admitted to Istanbul University, Istanbul Medical Faculty, Department of General Pediatrics between January 2006, and December 2013, with recurrent fever, abdominal pain, and arthritis/arthralgia, and skin eruptions.

A total of 1064 patients were admitted to our clinic within this time period. Five hundred sixty-two children (52.8 %) were diagnosed as having FMF and comprised our study group. The age range of the study group was 1.8-16 years.

**Fig. 1:**
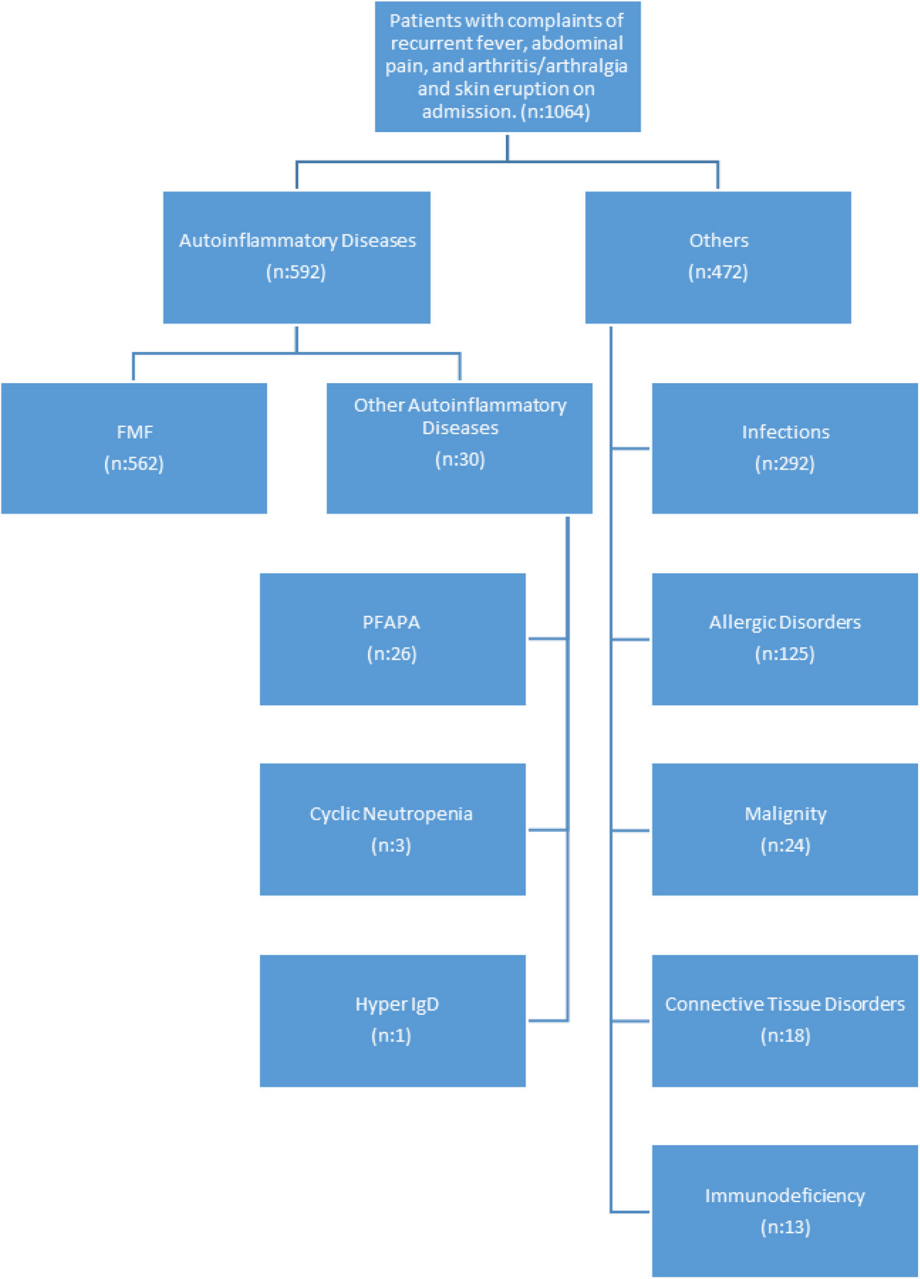
The distribution of patients admitted with complaints of recurrent fever, abdominal pain, and arthritis/arthralgia and skin eruption

The diagnosis of FMF was established in accordance with the Tel Hashomer Criteria [10] (Table 1). The remaining patients who had been admitted to our clinic were diagnosed as having infections (*n* = 292), allergic diseases (*n* = 125), malignancy (*n* = 24), connective tissue disease (*n* = 18), immunodeficiency syndromes (*n* = 13), PFAPA (*n* = 26), cyclic neutropenia (*n* = 3), and hyper IgD (*n* = 1) (Fig. 1).

**Table 1:** Tel-Hashomer diagnosis criteria [10]

Major criteria:
1-Recurrent febrile episodes associated with peritonitis, pleuritis or synovitis
2-Amyloidosis of AA-type without a predisposing disease
3-Favorable response to daily colchicine
Minor criteria:
1-Recurrent febrile episodes
2-Erysipelas-like erythema
3-Positive history of familial Mediterranean fever in a first degree relative
Definite Diagnosis: The diagnosis if definite is 2 major or 1 major + 2 minor criteria are met.
Probable diagnosis: The diagnosis is probable if 1 major + 1 minor criterion are met.

An acute episode of FMF was diagnosed with fever that lasted 1-3 days, accompanied by abdominal, chest, joint or muscle pain, erysipelas like skin eruption, arthritis and scrotal swelling, all of them disappearing spontaneously. The patients’ clinical and laboratory evaluation results were ‘normal’ between attacks. Severity of the disease was determined in accordance with the criteria used by Pras et al. [11]. (Table 2).

**Table 2:** Pras et al. severity scoring system for pediatric FMF [11]

	Variable	Score
Appearing of disease (year)	>11	2
	6-10	3
	<6	4
Frequency of attack on admission	<1	1
	1-2	2
	>2	3
Severity of arthritis	Acute	2
	Degenerative	3
Erysipelas-like erythema	present	3
Required dosage of colchicine (mg/day)	<1	1
	1-1.5	2
	2	3
	2^a^	4

Note: Score 3-5 Mild; 6-8 moderate; >9 severe disease. ^a^non-response to colchicine

The patients who had HSP underwent skin biopsy and immunoflorescence staining revealed deposition of IgA and C3 along small vessels.

The study was ethically approved by the ethics comitee within the Department of Pediatrics in our institution. Written and verbal consent was obtained from parents to use patient data for studies, but since this non-interventional study only reports our experience with patients managed according to the standard of care and patients did not undergo any tests that were requested solely for the purposes of obtaining data to include in this study, no separate consent for enrollment in the study was obtained.

### Laboratory evaluations

SAA was measured in accordance with the manufacturer’s instructions (Siemens Health Care Diagnostic Products GmbH, Marburg/Germany) by N Latex SAA kits in Behring Nephelometer Prospec machine. C-reactive protein (CRP) was quantified with immunoturbidimetric methods as per the manufacturer’s instructions (Roche Diagnostics GmbH, Sandhoffer Strasse, Mannheim/Germany) by C-Reactive protein Latex kits (COBAS INTEGRA/Cobas System) in Roche Hitachi Cobas C 501 analyzer.

The assay for proteinuria was a quantitative one based on a 24 h urine collection. It was repeated one month after initiation of treatment with colchicine.

### Gene/mutation analysis

For 592 of the 1064 patients who reported fever, arthralgia and skin eruptions, autoinflammatory diseases were considered as a diagnostic possibility and therefore, analysis of the pertinent gene mutations were ordered. 562 of these cases turned out to have mutations.

Patients provided 2 cc blood under appropriate clinical conditions. DNA subtracts were taken from the blood samples using a QIAamp DNA mini kit (QIAGEN, catalog no: 51306). An FMF Strip Assay (ViennaLab Labordiagnostika GmbH, Vienna, Austria) kit was used to determine the presence of an Mediterranean fever (MEFV) gene mutation in patients. For mutation analysis, exon 2 v 10 of the MEFV gene was amplified using multiplex polymerase chain reaction (PCR) [12]. Amplified products were hybridized with oligonucleotide probes. We screened for 12 MEFV gene mutations (E148Q in exon 2, P369S in exon 3, F479L in exon 5, M680I [G/C], M680I [G/A], 1692del, M694v, M6941, K695R, V726A, A744S, R761H in exon 10) using the FMF Strip Assay, Vienna Lab Diagnostics GmbH, Vienna, Austria. The mutations were called heterozygous, homozygous, and compound heterozygous.

Genetic mutation analysis was performed in Istanbul Medical Faculty Medical Biology laboratory. The white blood cell number, CRP, fibrinogen, erythrocyte sedimentation rate, SAA level, and protein amount in 24 h urine were investigated in the patients’ febrile periods. SAA level was also measured for a second time after treatment with colchicine in order to screen whether there was any influence with treatment.

Heterozygous patients were confirmed by sequence analyses.

Exon 2, 3, 5 and 10 was sequenced by using GML SeqFinder Sequencing System MEFV kit with Sanger sequence technology. The PCR conditions were as follows: Initial denaturation at 94 °C for 5 min, 35 cycles at 94 °C for 30 s and 58 °C for 45 s, 72 °C for 1 min, and a final extension at 72 °C for 5 min. The PCR products were visualized and collected with 2 % agarose gel electrophoresis, followed by purification using an Exo-SAP PCR purification Kit (UAB Corporation Cleveland, Ohio, USA). The product was sequenced in both strands initiating from the forward and the reverse primers used in the initial PCR and analysed on an ABI 3130 Automated DNA Sequencer (Applied Biosystems, Foster City, CA, USA). Further analysis was performed with SeqScape v2.6 and Sequencing Analysis 5.3.1 version programs [13].

### Statistical analysis

All analyses were performed using SPSS 21.0. All continuous variables were examined using the paired or unpaired *t*-test as appropriate. Non-continuous variables were examined using the Chi-square test. The ratio of mutation types and clinical findings were compared with variance analysis. A P value < 0.05 for the two-tailed *t*-test was considered statistically significant.

## Results

Five hundred sixty-two children (52.8 %) were diagnosed as having FMF among the 1064 patients who presented with recurrent fever, abdominal pain, and arthritis/arthralgia and skin eruption (Fig. 1).

### Patients and clinical findings

Two hundred eighty-seven (51.0 %) of the study population (*n* = 562) were female. The mean age was 6.9 ± 3.4 years (range, 1.8-11.6 years). The most common symptom was fever (97.3 %, *n* = 547). The other frequent symptoms were abdominal pain (543, 96.6 %) and arthritis/arthralgia 243/358 (43.2 %/63.7 %). Moreover, skin eruption was seen in 140 patients (24.9 %) and HSP was found in 105 patients (18.6 %). The patients’ detailed history and physical examination information are demonstrated in Table 3.

**Table 3:** The past medical history, response to colchicine, symptoms and mutation types of patients with FMF on admission

Variables	N	Percent
Sex		
Female	287	51
Male	275	49
Past Medical History		
FMF in family history	251	44.6
Appendectomy history	26	4. 6
Findings		
Fever	547	97.3
Abdominal pain	543	96.6
Arthralgia	358	63.7
Arthritis	243	43.2
Chest pain	229	40.7
Skin eruption	140	24.9
Henoch-Schonlein Purpura	105	18.6
Scrotal swelling	12	2.1
Response to Colchicine		
Complete	524	93.2
Non-response	38	6.8
Type of Mutation		
Heterozygote	252	44.8
Homozygote	228	40.5
Compound heterozygote	82	14.7

### Results of mutation analysis

The heterozygote mutation was positive in 252 patients (44.8 %), homozygote mutations were positive in 228 patients (40.5 %), and compound heterozygote mutations were positive in 82 patients (14.6 %). The total positivity of mutations was as following: M694V (*n* = 290, 51.6 %), E148Q (*n* = 114, 20.2 %), M680I (*n* = 64, 11.3 %), R202Q (*n* = 57, 10.1 %), V726A (*n* = 18, 3.2 %), P369S (*n* = 9, 1.6 %), K695R (*n* = 7, 1.2 %) and U726A (*n* = 3, 0.5 %). The most frequently seen mutations with sequence analysis in heterozygote mutations were Met694Val (*n* = 129, 51.1 %), pVal726Ala (*n* = 92, 36.5 %) and pAla744Ser (*n* = 31, 12.4 %). The most frequently seen mutations in compound heterozygote patients were M694V/E148Q (*n* = 32 39 %).

### The correlation of genetic mutations and symptoms

In total, 34 patients (6.04 %) were admitted with fever only, and their mean age was 2.1 ± 0.1 years. Of these patients, M694V was detected in 25 patients and R202Q homozygous mutation was found in 9 patients. The response to colchicine of all these patients was favorable.

Twenty-seven patients (4.8 %) who had no fever presented only with abdominal pain and their mean age was 4.5 ± 0.4 years. Among these patients, M694V was found in 20 patients and E148Q homozygote mutation was determined in 7 patients. The mean age of patients who were admitted with abdominal pain alone was significantly higher than those who presented only with fever (*p* = 0.04). Abdominal pain was significantly more frequent in patients with M694V and E148Q heterozygote mutation than in children with R202Q and M680I heterozygous mutations (*p* = 0.012).

228 of the 228 homozygous patients, 206 of the 252 heterozygous patients and 64 of the 82 compound heterozygous patients had their disease severity scored and evaluated to see if there are statistically significant differences depending on the type of mutation (Table 4).

**Table 4:** The distribution of severity of FMF according to genetic mutations and types (n:562)

	Homozygote mutations (n: 228; 40.5 %)							
	M694V	E148Q	M680I	R202Q	V726A	P369S	K695R	U726A
Mild	17	23	4	13	3			
Moderate	27	14	12	14	1			
Severe	70	12	10	6	2			
Total	114	49	26	33	6			
	Heterozygote mutations (n: 252; 44.8 %)							
	M694V	E148Q	M680I	R202Q	V726A	P369S	K695R	U726A
Mild	41	10	6	3	1	9	7	3
Moderate	57	9	8	4	2			
Severe	22	6	12	5	1			
Total	120	25	26	12	4	9	7	3
	Compound Heterozygote mutations (n: 82; 14.6 %)							
	M694V/E148Q	M694V/R202Q	M694V/M680I	E148Q/V726A				
Mild	12	3	1	5				
Moderate	15	2	2	3				
Severe	5	7	9					
Total	32	12	12	8				

The percentage of the clinically severe homozygote M694V mutations was 24.1 % (*n* = 70). This percentage was significantly higher than other homozygote mutations (*p* < 0.01).

Arthritis/arthralgia was significantly more common in patients with M694V and E148Q homozygous mutations (*p* = 0.014, *p* = 0.01, respectively).

Chest pain was more frequent in homozygous M694V (61,4 %) and heterozygous E148Q (25.6 %) mutation types (*p* = 0.04). Pericardial effusion was observed using echocardiography in 25 (10.9 %) of the 229 children with chest pain. The chest pain was unresponsive to colchicine in eight of these patients, consequently they were treated with methylprednisolone.

Skin eruptions were most commonly seen in homozygous E148Q mutations (*p* = 0.04). This was followed by heterozygous M694V and heterozygous M680I mutations.

Homozygous M694V (*n* = 34, 32.3 %) and homozygous E148Q (*n* = 23, 22 %) mutations were significantly more common in patients who also had HSP (*p* = 0.03). Detailed clinical findings and mutation analysis information are shown in Table 5.

**Table 5:** The correlation of clinical findings and mutation types in patients with FMF (n:562)

Mutation type	Fever	Abdominal pain	Arthralgia	Arthritis	Chest pain	HSP	Skin eruption	Response to colchicine	
								Complete	Non-response
	547	543	358	243	229	105	140	524	38
	n/%	n/%	n/%	n/%	n/%	n/%	n/%	n/%	n/%
M694V (290/562)	290/53	290/53.4	142/39.6	87/35.8	175/76.4	55/52.3	37/26.4	282/53.8	15/39.5
E148Q (114/562)	110/21.5	104/19.1	113/31.5	96/39.5	44/19.2	38/36.1	60/42.8	112/21.3	6/15.8
M680I (64/562)	60/10.9	62/11.4	40/11.1	35/14.4	8/3.4	7/6.6	21/15	59/11.2	12/31.5
R202Q (57/562)	55/10	55/10.1	47/13.1	18/7.4	2/0.8	5/4.7	5/3.5	55/10.4	5/13.2
V726A (18/562)	18/3.2	18/3.3	10/2.7	2/0.8	-	-	8/5.7	10/1.9	-
P369S (9/562)	9/1.6	8/1.4	3/0.8	2/0.8	-	-	9/6.4	2/0.3	-
K695R (7/562)	7/1.42	5/0.9	2/0.5	1/0.4	-	-	-	2/0.3	-
U726A (3/562)	1/0.1	1/0.1	1/0.2	1/0.4	-	-	-	2/0.3	-
M694V/R202Q (30/562)	30/5.5	30/5.5	11/3.1	18/7.4	11/4.8	14/13.3	16/11.4	30/5.7	-
M694V/E148Q (32/562)	30/5.5	32/5.9	24/6.7	17/7.0	12/5.2	6/5.7	8/5.7	32/6.1	
M694V/ M680I (12/562)	12/2.2	12/2.2	10/2.8	2/0.8	4/1.7	-	8/5.7	12/2.3	-
E148Q/V726A (8/562)	8/1.5	5/0.9	3/0.8	1/0.4	4/1.7	5/4.8	-	8/1.5	-

The most common febrile episodes were in patients with M694V homozygous mutations (number of attacks = 4.4 ± 1.6/month). This was followed by compound heterozygous M694V-M680I mutations (number of attacks = 3.6 ± 1.1/month) and M680I-E148Q mutations (number of attacks = 2.3 ± 1.1/month).

Fever (*n* = 30), abdominal pain (*n* = 32), arthritis (*n* = 17) and arthralgia (*n* = 24) were the most common symptoms in compound heterozygous M694V-E148Q mutations.

The patients with compound heterozygous M694V/R202Q (*n* = 30, 36.5 %) mutations had skin eruptions (*n* = 16, 53.3 %) and HSP (*n* = 14, 36.6 %) in addition to fever and abdominal pain.

Patients with a history of appendectomy (*n* = 26) carried M694V homozygous mutations (*n* = 16) and E148Q homozygous mutation (*n* = 10).

Some of (*n* = 12, 2.29 %) the colchicine responsive patients (*n* = 524) had diarrhea after one week. We reduced the dose and continued the therapy.

There was no difference in the clinical features and genotypes of the cases required hospitalization and those that were managed as outpatients. The M694 mutation was prevalent in both groups.

### Laboratory evaluations

The mean level of leucocytes was 17 580 ± 5571 mm^3^ (range, 5100-29 700) on admission and C-reactive protein was 64 ± 32.42 mg/dL (range, 6-245 mg/dL), erythrocyte sedimentation rate was 74.47 ± 32.24 mm/h (range, 8-132 mm/h), and fibrinogen was 463.24 ± 70.50 mg/dL (range, 298-875 mg/dL). Proteinuria was also detected in 106 patients (29.2 %) at an average of 854 ± 145 mg/L (Table 6). One month after initiation of treatment with colchicine, protein excretion at the end of the treatment month was found to be 236 ± 14.5 mg/dL, which was significantly lower (*p* < 0.05)

**Table 6:** Laboratory evaluations of patients with FMF

Variable	Mean ± SDS	Min-Max
Age (years)	8.58 ± 4.2	1 – 16
Leucocyte /mm ^3^	17.580 ± 5.571	5100 - 29700
CRP (mg/dL)	64.5 ± 32.42	6 - 245
ESR(mm/saat)	74.47 ± 32.24	8 - 132
Fibrinogen(mg/dL)	463.24 ± 70.50	298 - 875
Proteinuria (mg/L)	854 ± 145	520 - 2410
(positive in 106 patients)		

Among the patients with proteinuria, homozygous M694V mutations were determined in 75 patients, homozygous M680I was in 12, homozygous E202Q was in 9, homozygous E148Q was in 6 patients, and other mutations were detected in 4 patients.

The mean level of SAA on admission was 222.4 ± 100.0 mg/dL. This was found as 388.3 ± 35.6 in children with homozygote M694V mutations. This suggested that the SAA level in homozygous M694V mutation was significantly higher than in other mutations (Table 7). Moreover, all patients with homozygous mutations had higher levels of SAA compared with children with heterozygous and compound heterozygous mutations (*p* = 0.02 and *p* = 0.03, respectively). Patients who had a complete response to monthly colchicine therapy (*n* = 524) had an average 11.18 ± 2.6 mg/dL SAA level after treatment and there was no difference between mutation types.

**Table 7:** The response to colchicine therapy and serum level of SAA

	The response to colchicine therapy	
All of responsive	Before colchicine therapy	After colchicine therapy
	Mean ± SD mg/dL	Mean ± SD mg/dL
Homozygous	234.2 ± 22.3	14.1 ± 1.4
Heterozygous	154.3 ± 13.8	12.2 ± 1.3
Compound Heterozygous	182.1 ± 12.4	13.3 ± 1.2

Patients who had a partial response to monthly colchicine therapy (*n* = 38) had an average 156.34 ± 23.4 mg/dL SAA level. The level of SAA was significantly lower in complete responsive than in partial responsive (*p* = 0.001).

## Discussion

In this study we aimed to screen the correlation between genetic and phenotype of Turkish children who had been diagnosed as having FMF. Therefore, we investigated the 12 most frequent mutations and concomitant clinical and laboratory findings. The frequency of FMF among patients who were admitted with recurrent fever, abdominal pain, and arthritis/arthralgia and skin eruptions was 53.6 %. This result was similar to the study conducted by Ulgenalp et al. (50.5 %) [14].

The most common symptom was fever (97.3 %). The other frequent symptoms were abdominal pain (96.6 %) and arthritis/arthralgia (43.2 %/63.7 %). These clinical findings were mostly accompanied by M694V homozygous/heterozygous mutations. Similar to other studies, the most commonly seen mutation that accompanied clinical findings was M694V. Özelkaya et al. showed that the most frequent symptoms were abdominal pain (83.1 %) and fever (55 %). Other common findings reported are arthritis (17.1 %) and erysipelas-like erythema (7.7 %). It was also demonstrated that these clinical findings were mostly accompanied by M694V homozygote mutations [15–17].

Solak et al. reported the most common symptom to be abdominal pain, which was followed by fever, arthralgia, chest pain, and skin eruptions in a study of 165 patients. In this study, the most commonly seen mutations were M694V/I, E148Q and M680I [18]. Akar et al. reported that the most frequent mutations were M694V and M680I [19]. Giancane et al. reported that the E148Q variant is common, of unknown pathogenic significance and as the only MEFV variant does not support the diagnosis of FMF [20].

In our study, distinct from other studies, E148Q was the second most common mutation that accompanied fever, arthritis, and arthralgia. M680I was the third most common mutation and was found less frequently in patients with arthralgia/arthritis, but was seen more frquently in patients with skin eruptions.

Chest pain was most frequently seen in patients with M694V homozygous and E148Q heterozygous mutations. Similar to other studies, the M694V was the most commonly determined mutation in these patients.

Marek et al. researched the second other mutation apart from 12 mutations in patients with heterozygous MEFV gene mutations and demonstrated the second mutation in 25 % of patients [21]. We did not investigate second analysis in patients with heterozygous mutations.

The mean age of patients who only had fever on admission was significantly lower than patients who presented with abdominal pain alone. It has been reported that fever is a common finding in younger patients; however, serositis, skin eruptions and other findings are more often seen in older patients with FMF [22]. Padeh et al. also stated that the first symptom of the disease might be fever and serositis may develop later in the course [23]. MEFV gene mutations can be found in patients with HSP. FMF has been reported more frequently in patients who had HSP than in the general population. MEFV mutations may be related to HSP susceptibility in children [24]. Salah et al. found heterozygous mutations of V726A and E148Q to be the most common in children who also had HSP [25]. In contrast, we found homozygous mutations of M694V and E148Q to be the most common. Dogan et al. found that 11 of their 76 patients (14.4 %) were heterozygous, and 5 patients (6.6 %) were homozygous and 2 were compound heterozygous [26]. Altug et al., found that 18 of their 68 patients with HSP had MEFV mutations, 15 of them being heterozygous [27].

Our study included more patients who were first diagnosed with FMF and later went on to have HSP. This could be the reason why our study had more homozygous mutations in contrast to the findings of the three studies mentioned above [27].

In our study, in patients who had been previously diagnosed as having FMF and presented with findings pertinent to HSP, we found that the most common mutation of the 105 patients was M694V (*n* = 55, 52.3 %); the second most common was E148Q (*n* = 28, 26.6 %). Homozygous M694V and E148Q mutations were particularly common (*p* = 0.03).

FMF strip assay is a fast screening test; the most common major 12 mutations with known clinical meaning can be investigated. Screening can investigate only 12 mutations, whereas sequencing gives a result of all changes of exons 2,3,5 and 10 on MEFV gene that can be reviewed. More detailed sequencing tehcniques can reveal that clinically diagnosed patients who had none of the 12 most common mutations may actually have some of the less common mutations or novel mutations.

Proteinuria was reported to be positive most commonly with the M694V mutation and less frequent with E148Q [28].

Serum Amyloid A levels on admission with homozygous M694V mutation were significantly higher than with other mutations such as heterozygous and compound heterozygous mutations. Similar to the study of Berkun et al., the SAA level was higher in patients with homozygous M694V mutation [29]. According to a study conducted by Duzova et al., SAA was higher with homozygous and compound heterozygous mutations [30]. There was no significant difference in SAA level between mutation types in children with complete response. The level of SAA was significantly lower in patients who were completely responsive to colchicine than in partially responsive patients [31]. Similar to our study, the authors emphasized that administration of colchicine might be effective in diminishing the SAA level [31]. In our study, we showed a decrease in level of SAA in patients who had a complete response to colchicine therapy. The dose of colchicine was not increased.

A retrospective case series review was conducted of all MEFV gene mutation testing completed at UCLA Clinical Molecular Diagnostic Laboratory by Ong et al. to correlate specific genotypes with adverse phenotypes of affected populations residing in the Western United States, between February 2002, and February 2012. This was followed by a clinical chart review of all subjects who either had a single or double mutation. All 12 common mutations in the MEFV gene were analyzed and the M694V variant was found to be associated with an adverse FMF clinical outcome in the Armenian-American population, which manifested as earlier onset of disease, increased severity of disease, and renal amyloidosis [32].

In our study, M694V was found to be the most common mutation and it heralds more proteinuria and a more severe clinical course.

## Conclusion

In summary, the frequency of FMF was 52.8 %. Fever was the most frequent symptom. The most common mutation was M694V. The mean age of patients who were admitted with abdominal pain alone was significantly higher than patients who presented with fever alone. Abdominal pain was significantly more frequent in patients with M694V and E148Q heterozygous mutations than children with R202Q and M680I heterozygous mutations. Arthritis/arthralgia were significantly more common in patients with homozygous M694V and E148Q mutations. Chest pain was more frequent with homozygous M694V (61.4 %) and heterozygous E148Q (25.6 %) mutations (*p* = 0.04). In 25 of the 229 children (10.9 %) with chest pain, pericardial effusion was documented at echocardiography. Skin eruption was most commonly seen in homozygous E148Q mutations. This was followed by heterozygous M694V and heterozygous M680I mutations. Homozygous M694V (*n* = 34, 32.3 %) and E148Q (*n* = 23, 22 %) mutations were significantly more frequent in patients with HSP (*p* = 0.03). Arthritis (*n* = 17, 7.0 %) and arthralgia (*n* = 24, 6.7 %) were seen more frequently in patients with compound heterozygous M694V/E148Q mutations, whereas M694V/R202Q coexistence caused skin eruptions (*n* = 16, 11.4 %) more frequently. The most frequent occurence of febrile episodes were in patients with M694V homozygous mutations (number of attacks = 4.4 ± 1.6/month). Patients with a history of appendectomy (*n* = 26) carried homozygous M694V (*n* = 16) and E148Q (*n* = 10) mutations. Diarrhea developed after colchicine therapy in 0.22 % of patients. The ratio of the clinically-severe patients was 24.1 % (*n* = 70) in children with homozygous M694V mutations. This ratio was significantly higher than other homozygous mutations. Proteinuria was detected most frequently in patients with homozygous M694V mutations. The SAA level in homozygous M694V mutations was significantly higher than in other mutations.

Our study was comprehensive and has demonstrated the correlation between the genotype and the clinical characteristics of FMF in Turkish children. Patients were classified and analyzed according to clinical, laboratory, and genotype features. However, a major limitation of our study is its retrospective design and the lack of a demographic analysis due to insufficient information about the place of birth of the patients.
